# Combining Point-of-Care Diagnostics and Internet of Medical Things (IoMT) to Combat the COVID-19 Pandemic

**DOI:** 10.3390/diagnostics10040224

**Published:** 2020-04-16

**Authors:** Ting Yang, Mattia Gentile, Ching-Fen Shen, Chao-Min Cheng

**Affiliations:** 1 Institute of Biomedical Engineering, National Tsing Hua University, Hsinchu 300, Taiwan; tinayang0402@gmail.com; 2 Department of Medical Genetics, Di Venere Hospital, ASL BARI, 701 Bari, Italy; mattia.gentile@asl.bari.it; 3 Department of Pediatrics, National Cheng Kung University Hospital, College of Medicine, National Cheng Kung University, Tainan 704, Taiwan

The current standard testing method for screening coronavirus disease 2019 (COVID-19) is through reverse real-time PCR assay (rRT-PCR), a common molecular-based assay that requires an average of four to six hours to provide results. Although this tool is widely used, it relies on a well-equipped laboratory, trained specialists, and is time-consuming. This limits the number of tests that can be performed. As the COVID-19 outbreak becomes less and less controllable, millions of lives have been threatened, resulting in breakdown of medical systems and considerable worldwide panic. It seems quite clear that rRT-PCR based testing is not useful in the control of the disease epidemic due to the high rate of asymptomatic cases: recent data on a small sample (*n* = 60) of healthy blood donors in Castiglione D’Adda (epicenter area of Italy), indicated that over 70% of cases had SARS-CoV-2 antibodies [1]. Provision of a suitable COVID-19 diagnostic platform could save lives and alleviate the pressure on front-line healthcare workers and healthcare systems.

## 1. Rapid Diagnostic Tools for COVID-19

As an alternative to the currently limited COVID-19 diagnostic approaches, point-of-care (POC) devices employing lateral flow immunoassay (LFIA) technology have been developed to detect COVID-19 in human serum [2]. IgG and IgM antibodies against SARS-CoV-2 can be detected in human blood after COVID-19 infection. An examination of the levels of these antibodies can provide information on the progress and stages of viral infection. As the number of confirmed worldwide cases rises, several POC LFIA devices with the capacity to simultaneously detect IgG and IgM levels have been developed and now serve as screening tools for COVID-19; these tools are being used to fill the urgent need for additional and rapid diagnostic tools. Examples of LFIA devices for COVID-19 diagnosis were mainly based on the detection of IgG/IgM antibodies. The CE certified coronavirus (COVID-19) rapid test developed by Sure Biotech, USA can test the IgG/IgM antibodies with either whole blood or serum within 20 minutes [3]. Other promising LFIA diagnostics for either whole blood or serum tests are the GenBody COVID-19 IgM/IgG device conducted by GenBody, Korea; the COVID-19 IgM/IgG rapid test launched by BioMedomics, USA; and the Coronavirus COVID-19 Test—Home Self Test Kit produced by The Hive Pharmacy, UK. While tests for examining COVID-19-associated antibodies in blood are already in use, additional approaches have been developed. Tan et al., have found higher positive test results using specimens such as nucleocapsid (N) or spike (S) proteins collected via nasal or pharyngeal swabs (potentially used at home) [4]. As of March 28, 2020, several possible POC diagnostic tools working with samples collected from swabs have received emergency use authorization from the FDA, extending the possible options of sampling tools.

## 2. Concept of the Internet of Medical Things (IoMT)

As COVID-19 spreads more and more rapidly, cities are facing sudden lockdown measures, forcing nearly ten billion to self-quarantine at home. The demand for necessary medical supplies and equipment has been high and is likely to outpace the capacity for rapid, but vitally necessary replenishment. Citizens and potential patients must leave their homes to seek medical help, which pokes a hole in isolation and quarantine efforts that threatens disease control. Additionally, the shortage of isolation wards and proper medical devices has prompted the medical community to encourage those with mild or suspected symptoms to remain at home. Clearly an alternative, home-based diagnostic test would offer an invaluable solution to fill this urgent and unmet need.

**Figure 1:**
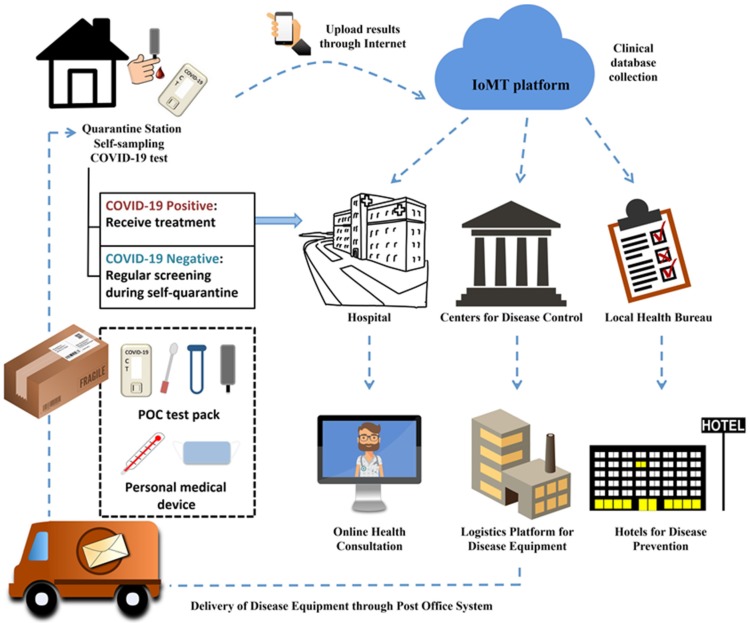
Schematic of the medical ecosystem concept for combating coronavirus disease 2019 (COVID-19): combined implementation of point-of-care (POC) diagnostics and the Internet of Medical Things (IoMT).

The Internet of Medical Things (IoMT), is the extended, healthcare-specific version of the Internet of Things (IoT) [5]. Applied to the current crisis, it could be used to create a medical platform to help patients receive proper healthcare at home and establish a comprehensive disease management database for government and healthcare organizations, as shown in Figure 1. People with mild symptoms could obtain diagnostic and healthcare equipment (protective masks, thermometers, medications, POC COVID-19 kits for diagnosing and monitoring infection). Patients could upload their health status regularly to the IoMT platform (clinical cloud storage) via the internet, and their information could be transferred to nearby hospitals, the Center for Disease Control (CDC), and state and local health bureaus. Hospitals could subsequently offer online health consultations based on each patient’s health condition, and the government (the CDC, and local and state health bureaus) could allocate equipment and designate quarantine stations (hotels or centralized quarantine facilities) if necessary. Using the IoMT platform, people could dynamically monitor their disease status and receive proper medical needs without spreading the virus to others. This would reduce national health costs, mitigate the stresses of medical device shortage, and provide a systemic database that would allow the government to adequately monitor disease spread, appropriately distribute supplies, and implement emergency strategies.

## 3. Medical Ecosystem for Highly Infectious Diseases

In the past, the development of POC devices for infectious diseases was mainly dedicated to low-resource settings [6]. However, they are also well suited for use in mitigating highly contagious disease outbreaks such as the current COVID-19 pandemic. The necessary strategy of self-quarantine during such conditions urges the development, production, and implementation of a supportive ecosystem that incorporates home-screening, POC devices, and the IoMT for disease diagnosis and monitoring [7].

The pandemic we are currently battling is rapidly straining the healthcare industry toward its breaking point; already, clinical centers are overwhelmed with confirmed cases and suspected cases awaiting diagnostic confirmation. The demands for these services are on the rise, but there is a shortage of diagnostic devices and supplies that limits the reach of patient care and simultaneously puts mission-critical healthcare workers on the front lines at greater risk. A strong, supportive medical ecosystem could play a highly impactful role in mitigating this crisis and saving lives.

There is a clear need for a more comprehensive medical platform for fighting highly contagious diseases. Limited clinical resources and the necessary implementation of self-quarantine procedures urge the development and implementation of POC, home-based diagnostic devices that alleviate testing and monitoring burdens and enforce stay-at-home protocols. Combining such an approach with the IoMT would provide information to healthcare facilities for the development of suitable patient care protocols, and to government organizations for the appropriate allocation of equipment and supplies. This combined approach could be used to save countless lives, protect strained economies, and create a blueprint for more effectively combatting future threats.
